# Efficacy of acupuncture-based traditional Chinese medicine therapies for chronic fatigue syndrome: a systematic review and meta-analysis

**DOI:** 10.3389/fneur.2026.1860788

**Published:** 2026-06-24

**Authors:** Yanbing Li, Gaozhe Liang, Tingting Xiao, Lei Xu

**Affiliations:** The Second Affiliated Hospital of Guangdong Medical University, Zhanjiang, China

**Keywords:** acupuncture, chronic fatigue syndrome, Meta- analysis, systematic review, traditional Chinese medicine therapies

## Abstract

**Objective:**

To systematically review the available evidence on acupuncture-related therapies for chronic fatigue syndrome (CFS), with cautious interpretation due to the limited number and heterogeneity of studies.

**Methods:**

PubMed, Embase, Web of Science, and Cochrane Library were systematically searched for randomized controlled trials (RCTs) on acupuncture-related therapies for CFS from database inception to April 1, 2026. Two researchers independently performed literature screening, data extraction, and risk of bias assessment. The methodological quality of the included studies was evaluated using the Cochrane RoB 2 tool for RCTs. Meta-analysis was conducted using R software. Continuous outcomes were expressed as standardized mean difference (SMD) with 95% confidence interval (CI), and binary outcomes as risk ratio (RR) with 95% CI. Primary outcomes included overall fatigue, physical fatigue, mental fatigue, total response rate, and incidence of adverse events.

**Results:**

A total of 8 RCTs involving 715 patients were included. Risk of bias assessment showed that one study had a high overall risk of bias, and seven studies raised “some concerns.” Meta-analysis results indicated that acupuncture-related therapies were superior to control interventions in improving overall fatigue, with a statistically significant difference (SMD = −1.31, 95% CI: −2.16 to −0.47, *p* = 0.002), although heterogeneity was high (I^2^ = 91.7%). They were also superior to controls in improving physical fatigue (SMD = −0.52, 95% CI: −0.88 to −0.16, *p* = 0.0048; I^2^ = 63.8%) and mental fatigue (SMD = −0.59, 95% CI: −0.93 to −0.26, *p* = 0.0006; I^2^ = 56.5%). The total response rate was higher in the intervention group than in the control group, with a statistically significant difference (RR = 1.22, 95% CI: 1.07 to 1.40, *p* = 0.004; I^2^ = 0.0%). Safety analysis showed no statistically significant difference in the risk of adverse events between the intervention and control groups (RR = 2.26, 95% CI: 0.76 to 6.76, *p* = 0.144; I^2^ = 0.0%).

**Conclusion:**

Current limited evidence indicates that acupuncture-related therapies may have some beneficial effects on overall, physical, and mental fatigue in patients with CFS, and may increase response rates; however, these findings should be interpreted cautiously due to small sample sizes, moderate risk of bias, and heterogeneity among studies. No explicit safety hazards have been confirmed. And further validation by more high-quality RCTs is needed.

**Systematic review registration:**

This systematic review protocol was preregistered on the Open Science Framework (OSF) (https://osf.io. Registration DOI: 10.17605/OSF.IO/KJ67M).

## 1 Introduction

Chronic fatigue syndrome (CFS), often co-designated with myalgic encephalomyelitis (ME) as ME/CFS, is a chronic disease of unclear etiology that involves multiple systems and leads to substantial functional impairment (1–3). Its main clinical features are persistent or recurrent pathological fatigue, usually lasting more than 6 months and not fully relieved by rest; in addition, patients frequently experience post-exertional malaise, non-restorative sleep, cognitive decline, pain, and autonomic dysfunction (4, 5). Given the current lack of universally accepted specific biomarkers or objective diagnostic indicators, the clinical diagnosis of CFS/ME still relies mainly on symptom-based criteria and exclusionary assessment (6, 7). This situation not only increases the complexity of clinical recognition and classification but also contributes, to some extent, to inconsistencies in case definitions, study populations, and outcome evaluation methods across different studies (8).

Current epidemiological data indicate that CFS/ME carries a considerable disease burden. A systematic review showed that the prevalence varies according to case definitions, survey methods, and study settings; when the CDC-1994 criteria are used, the reported prevalence is relatively higher, and the risk of illness is generally higher in women than in men (9, 10). Moreover, CFS/ME persistently affects patients’ learning, work, and social functioning, and some patients remain significantly limited in their activities over the long term, with important impacts on individual quality of life and healthcare resource utilization (11). Therefore, CFS/ME is no longer regarded as a mere subjective fatigue state, but rather as a complex chronic disease with clear clinical burden and public health significance (8, 11).

Although research on CFS/ME has increased in recent years, its pathophysiological mechanisms remain incompletely understood. It is now generally believed that multiple factors—including immune and inflammatory abnormalities, neuroinflammation, autonomic nervous system imbalance, energy metabolism disorders, and mitochondrial dysfunction—may jointly participate in the onset and development of the disease (12, 13). In particular, core manifestations such as post-exertional worsening of symptoms, cognitive decline, and orthostatic symptoms may be closely related to dysregulation of the neuro-immune-endocrine network (14, 15). However, because of the strong heterogeneity of the disease itself, the key mechanisms suggested by different studies are not entirely consistent, which makes it difficult to establish a unified pathological model and standardized intervention pathway (16).

In terms of treatment, there is currently no universally accepted specific therapy for CFS/ME, and clinical management mainly focuses on symptom relief, maintenance of function, and individualized supportive care. For this reason, the search for safe, acceptable adjunctive treatments that may help improve core symptoms has become an important research direction in this field. Acupuncture-related therapies, as commonly used non-pharmacological interventions, have attracted increasing attention for their application in fatigue-related and chronic functional diseases. Nevertheless, the efficacy of acupuncture for CFS still needs to be further clarified through more rigorous evidence-based research. Based on this, the present study included RCTs of acupuncture-related therapies for CFS, and through a systematic review and meta-analysis comprehensively evaluated their effects on overall fatigue, physical fatigue, mental fatigue, clinical effectiveness, and safety, with the aim of providing a basis for clinical practice and future research.

## 2 Methods

### 2.1 Study design

The present study was conducted as a systematic review and meta-analysis to investigate the clinical effectiveness and safety of acupuncture-related treatments for CFS. The study was prospectively registered on INPLASY (Registration number: INSPLASY202650138; https://www.inplasy.com) and was designed, implemented, and reported in accordance with the PRISMA 2020 statement. The study population was restricted to patients with a definite diagnosis of CFS, and the study type was restricted to RCTs to improve the internal validity of the evidence and the comparability of results. For studies that also included other types of chronic fatigue, only data from the subgroup meeting CFS diagnostic criteria were extracted; among them, the study by Kim et al. (17) contributed only the CFS subgroup data.

### 2.2 Inclusion and exclusion criteria

The inclusion and exclusion criteria were developed according to the PICOS principle. Included studies had to meet the following criteria: participants were patients with CFS, with no restrictions on age, sex, ethnicity, or geographic region; the study design was an RCT; the experimental group received acupuncture-related therapies, including acupuncture, electroacupuncture, time-specific acupuncture, special needling techniques, moxibustion, ginger-separated moxibustion, and acupuncture combined with Chinese herbal medicine or conventional treatment; the control group received conventional treatment, pharmacotherapy, lifestyle intervention, non-time-specific acupuncture, non-acupoint acupuncture, Chinese herbal medicine alone, or other non-acupuncture treatments; and at least one fatigue-related outcome was reported, such as the Fatigue Severity Scale (FSS), Fatigue Scale-14 (FS-14), Chalder Fatigue Scale, Bell CFS score, or clinical response rate. Exclusion criteria were: non-RCTs, case reports, case series, reviews, systematic reviews, meta-analyses, conference abstracts, basic research, and animal studies; duplicate publications; studies in which participants were not CFS patients or CFS patient data could not be extracted; interventions or control measures that did not meet the predefined criteria; and studies that did not report extractable outcome data or whose data could not be converted or pooled.

### 2.3 Information sources and search strategy

We conducted a systematic literature search to identify relevant RCTs on acupuncture-related interventions for CFS. Four electronic databases were searched: PubMed, Embase, Web of Science, and the Cochrane Library. The search timeframe spanned from the launch date of each database up to April 1, 2026. To minimize the chance of missing eligible studies, we also examined the reference lists of the included articles and those of relevant reviews. The search strategy was structured around three main elements: study population, intervention type, and study design. Both controlled vocabulary (subject headings) and free-text terms were used. The main search terms included “chronic fatigue syndrome”, “myalgic encephalomyelitis”, “fatigue syndrome, chronic”, “acupuncture”, “electroacupuncture”, and “moxibustion.” All database-specific strategies were adapted to fit syntax and indexing rules. This comprehensive documentation ensures reproducibility across databases.

Search strategies were developed for all four databases (PubMed, Embase, Web of Science, and Cochrane Library) and are provided in Supplementary Table S1. The PubMed search strategy is exemplified as follows: (“Chronic Fatigue Syndrome” [Mesh] OR “chronic fatigue syndrome” OR “myalgic encephalomyelitis” OR “fatigue syndrome, chronic”) AND (“Acupuncture Therapy” [Mesh] OR acupuncture OR electroacupuncture OR moxibustion) AND (randomized controlled trial OR randomized OR randomly OR trial). If the present study restricted its search to English databases or imposed language limits, this should be specified during finalization to maintain consistency between methods and results.

### 2.4 Study selection and data acquisition

To manage the identified literature and eliminate redundancies, all records were imported into EndNote. The screening process involved two independent investigators who initially assessed titles and abstracts against established eligibility benchmarks. For citations deemed potentially relevant, the full texts were retrieved and scrutinized based on rigorous inclusion and exclusion criteria. Any disagreements between the primary reviewers were resolved through internal deliberation; if a consensus could not be reached, a third senior researcher provided an arbitration decision. The entire selection workflow was mapped in accordance with the PRISMA 2020 framework.

Regarding data extraction, two reviewers utilized a standardized template to independently gather information from the final pool of studies. The consistency of the extracted datasets was ensured through meticulous cross-verification. Inter-rater reliability for data extraction and risk-of-bias assessment was quantified using Cohen’s kappa (*κ* = 0.87 for selection, κ = 0.81 for quality assessment), indicating substantial agreement between reviewers. Captured variables included: bibliographic data (first author, publication year, and geographic location), methodological details (study design, sample size, and diagnostic criteria), participant characteristics, intervention specifics (dosage, duration, and follow-up), and outcomes (dropout rates, adverse effects, and risk of bias metrics). For trials with multiple arms, we focused on the intervention and control groups most aligned with our research objectives to prevent redundant participant counting. In cases where outcomes were reported across various intervals, data from the treatment’s conclusion were prioritized for primary analysis, while subsequent follow-up data served as supplementary descriptive evidence.

### 2.5 Quality assessment of included studies

The methodological rigor of the randomized trials was scrutinized using the Cochrane Risk of Bias 2 (RoB 2) instrument. This assessment encompassed five distinct domains of potential bias: the randomization process, deviations from intended interventions, missing outcome data, measurement of outcomes, and selection of the reported results. Following the RoB 2 algorithm, each domain was categorized into three levels: low risk, some concerns, or high risk, culminating in an overall bias determination for each study. To ensure the reliability of the findings, two authors performed the assessments independently. Discrepancies between the primary evaluators were settled through consensus-building discussions; where necessary, a third author was consulted to reach a definitive consensus.

### 2.6 Data synthesis and statistical methods

All computational tasks were performed using R software. We expressed results for binary outcomes (e.g., incidence of adverse events) as RRs, while continuous outcomes from different scales (FSS, FS-14, etc.) were aggregated using SMDs; both measures were reported with 95% CIs.

To address statistical heterogeneity, we employed the I^2^ statistic and the χ^2^ test (using a 0.10 significance threshold). Given the clinical diversity of acupuncture interventions across the literature, we conducted random-effects meta-analysis using the R package ‘meta’ (version 6.2–0). Pooled effects were estimated with the restricted maximum likelihood (REML) method; sensitivity analyses using the DerSimonian-Laird method were also performed to confirm robustness of results. In instances of substantial heterogeneity (I^2^ exceeding 50%), exploratory subgroup analyses based on intervention categories were conducted. For trials with more than two arms, we ensured that control subjects were only included once in the analysis. Significance for all two-sided tests was established at *p* < 0.05.

## 3 Results

### 3.1 Identification of eligible studies

**Figure 1:**
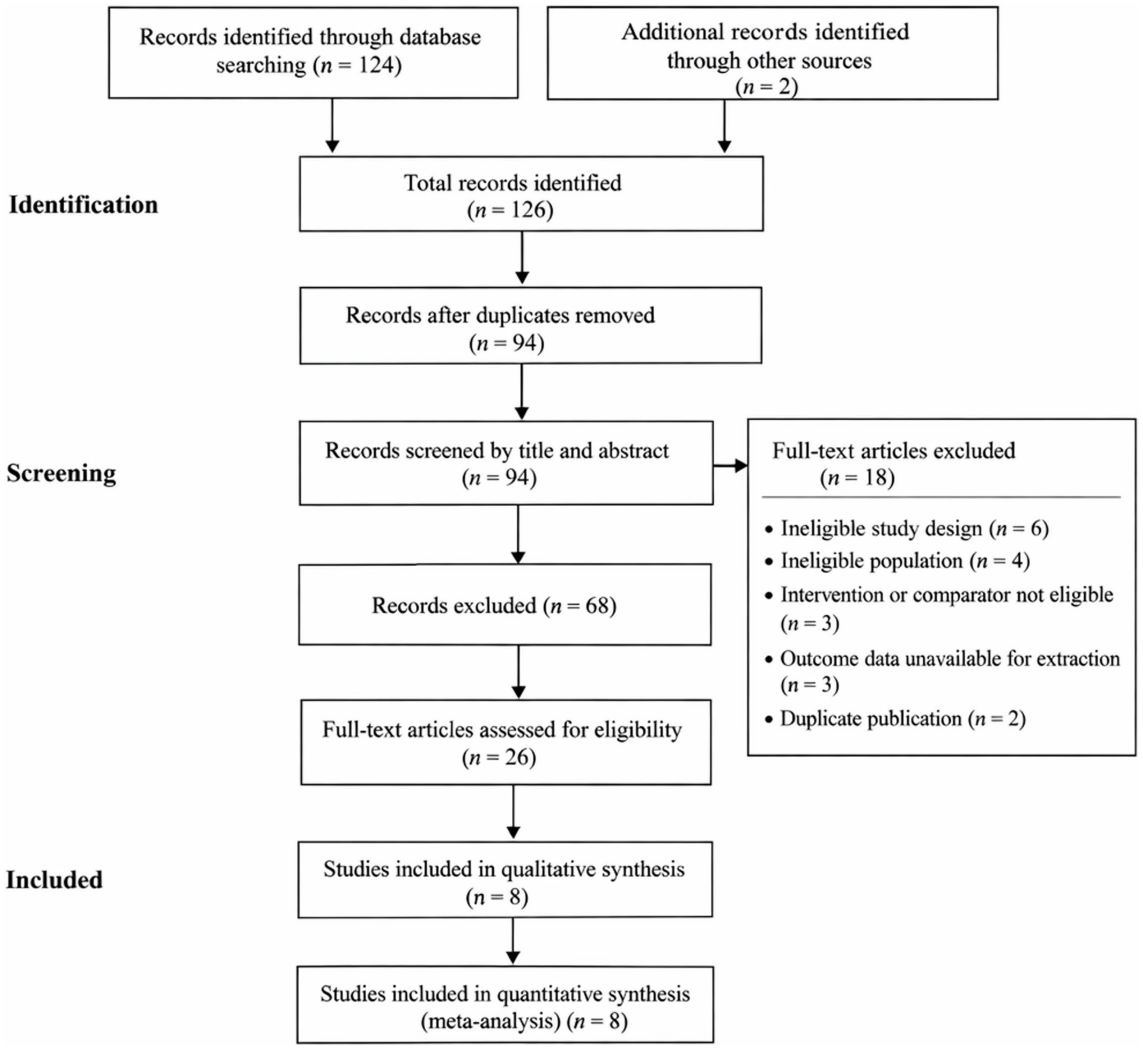
Flow diagram of the literature screening process.

From an initial pool of 126 retrieved records (124 from database searches and 2 from other origins), 94 remained following deduplication. A primary screening of titles and abstracts resulted in the removal of 68 ineligible citations. We then retrieved and scrutinized the full texts of the 26 remaining candidate articles. During this final appraisal, 18 studies were rejected based on specific grounds: inappropriate methodology (*n* = 6), participant non-eligibility (*n* = 4), protocol deviations (*n* = 3), lack of extractable data (*n* = 3), and duplicate reporting (*n* = 2). Consequently, 8 trials were deemed eligible for final inclusion in the qualitative and meta-analytic synthesis (Figure 1).

### 3.2 Basic characteristics of included studies

Eight RCTs published between 2010 and 2021 were included. Seven were conducted in China, and one in Korea, involving a total of 715 patients. All participants had CFS, and some studies further restricted inclusion by TCM pattern differentiation. Interventions mainly included acupuncture, moxibustion, and acupuncture combined with herbal medicine. Control interventions included conventional treatment, pharmacotherapy, lifestyle intervention, non-time-specific acupuncture, non-acupoint acupuncture, and herbal medicine alone. Treatment duration ranged from 14 days to 4 weeks. Most studies did not report post-treatment follow-up; only one study reported follow-up at 5 and 13 weeks. Primary outcome measures included FSS, FS-14, Chalder Fatigue Scale, Bell score, and total response rate (Table 1).

**Table 1:** Basic characteristics of the included studies.

Study	Country	Study design	Study population	Sample size (*n*)	Intervention	Control	Course	Follow-up	Primary outcome
Kim 2015 (17)	Korea	Multicenter, open-label, three-arm parallel RCT	Patients with CFS and ICF; only CFS subgroup extracted for this meta-analysis	Total 150; CFS subgroup 63	Body acupuncture/Four-constitution acupuncture combined with conventional treatment	Conventional treatment	4 weeks	5 weeks, 13 weeks	FSS
Xu 2019 (18)	China	Single-center, parallel RCT	CFS patients, aged 18–55 years	68	Acupuncture	Oryzanol + Vitamin B1 + rest and exercise	4 weeks	None	FS-14
Ling 2013 (19)	China	Parallel RCT	CFS patients with Qi deficiency pattern	80	Time-specific acupuncture	Non-time-specific acupuncture	20 days	None	Fatigue scale, total response rate
Chen XH 2010 (20)	China	RCT	CFS patients with Qi-Yin deficiency pattern	90	Acupuncture	Shenmai injection	14 days	Immediate assessment	FS scale
Qi 2017 (30)	China	Parallel RCT	CFS patients with liver-depression and spleen-deficiency pattern	60	Chinese herbal medicine + acupuncture	Chinese herbal medicine alone	30 days	None	FS-14
Xu 2012 (31)	China	Parallel RCT	CFS patients	72	Coiling dragon needling + moving cupping	Prednisone	14 days	None	Bell score, total response rate
Lu 2014 (32)	China	Three-arm parallel RCT	CFS patients, aged 18–55 years	133	Acupuncture / Acupuncture plus moxibustion	Non-acupoint acupuncture	20 days	None	Chalder Fatigue Scale
Lin 2021 (33)	China	Parallel RCT	CFS patients, aged 18–60 years	57	Ginger-separated moxibustion + lifestyle intervention	Lifestyle intervention alone	4 weeks	None	FS-14

### 3.3 Risk of bias assessment

**Figure 2:**
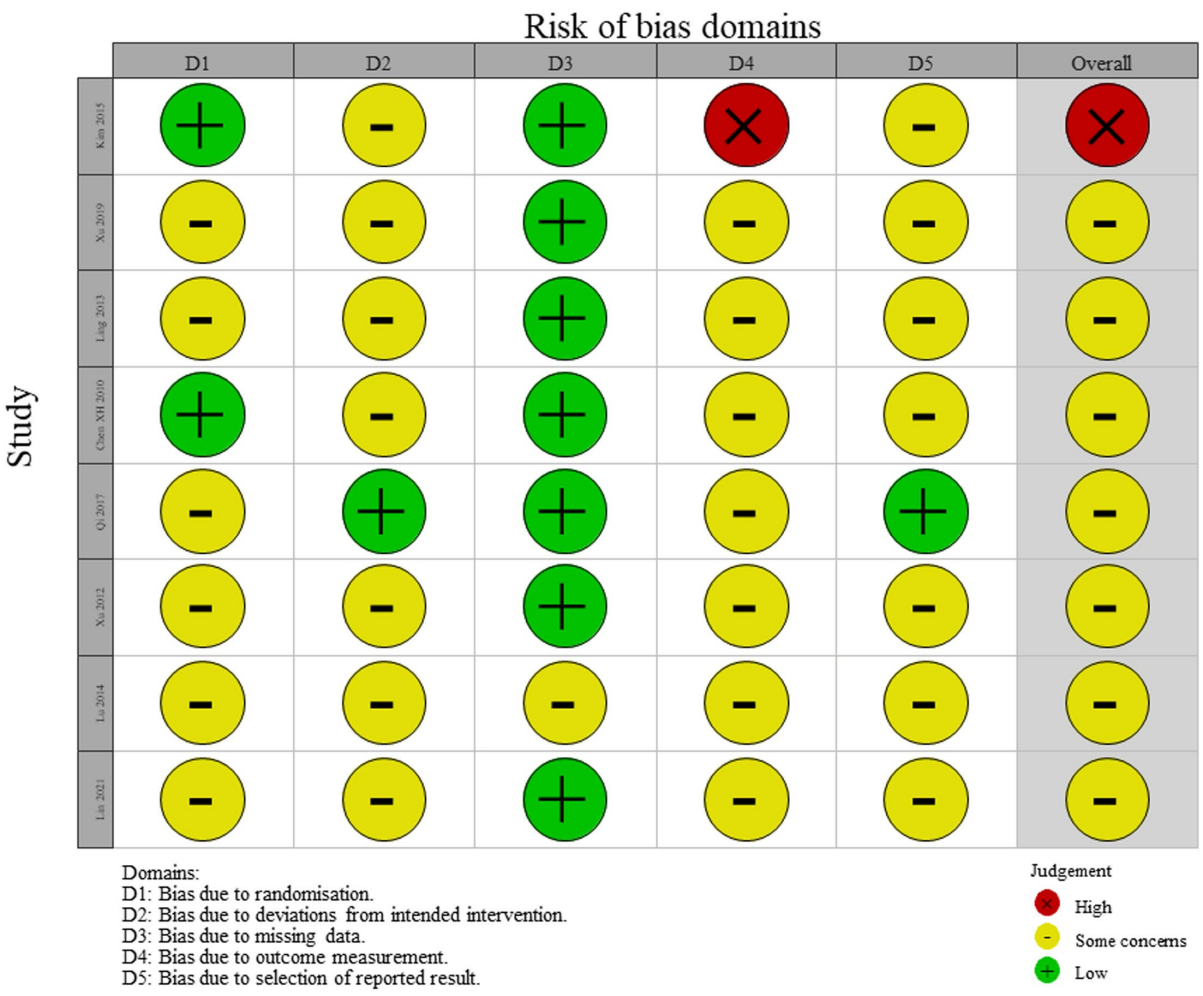
Risk of bias assessments across domains.

We applied the RoB 2 tool to evaluate the methodological quality of the eight RCTs included in this review. Among the 8 included studies, only one was at low overall risk of bias, seven had ‘some concerns’, and one was at high risk. Major methodological issues included insufficient description of random sequence generation and allocation concealment in six studies, lack of blinding in seven studies (only one study implemented blinding, none blinded outcome assessors), and absence of pre-registration in six studies, raising concerns of selective reporting. When examining the randomization process specifically, two studies received a rating of low risk. For the other six studies, the judgment was “some concerns,” primarily because the descriptions provided for random sequence generation or allocation concealment were insufficient. For deviations from intended interventions, only 1 study was at low risk, and the others were judged as “some concerns” mainly because blinding was not implemented. For missing outcome data, 7 studies were at low risk and 1 study raised “some concerns.” For outcome measurement, 1 study was at high risk, and the remaining studies raised “some concerns,” mainly related to the subjective nature of the outcome measures and inadequate blinding. For selective reporting, 2 studies were at low risk, and the others raised “some concerns” because of the lack of pre-registration or study protocol information. Overall, these methodological flaws may have led to an overestimation of effect sizes, and the overall risk of bias can be considered moderate. The risk of bias assessments across domains are shown in Figure 2.

### 3.4 Meta-analysis findings

#### 3.4.1 Analysis of overall fatigue

A total of 551 subjects (intervention: 312; control: 239) across nine comparisons were included in the overall fatigue analysis. Due to significant inter-study heterogeneity (I^2^ = 91.7%), a random-effects framework was employed. The synthetic results indicated a superior fatigue-reducing effect of acupuncture interventions compared to control treatments (SMD = −1.31, 95% CI: −2.16 to −0.47, *p* = 0.002); however, this pooled estimate should be interpreted cautiously due to the extremely high heterogeneity.

**Figure 3:**
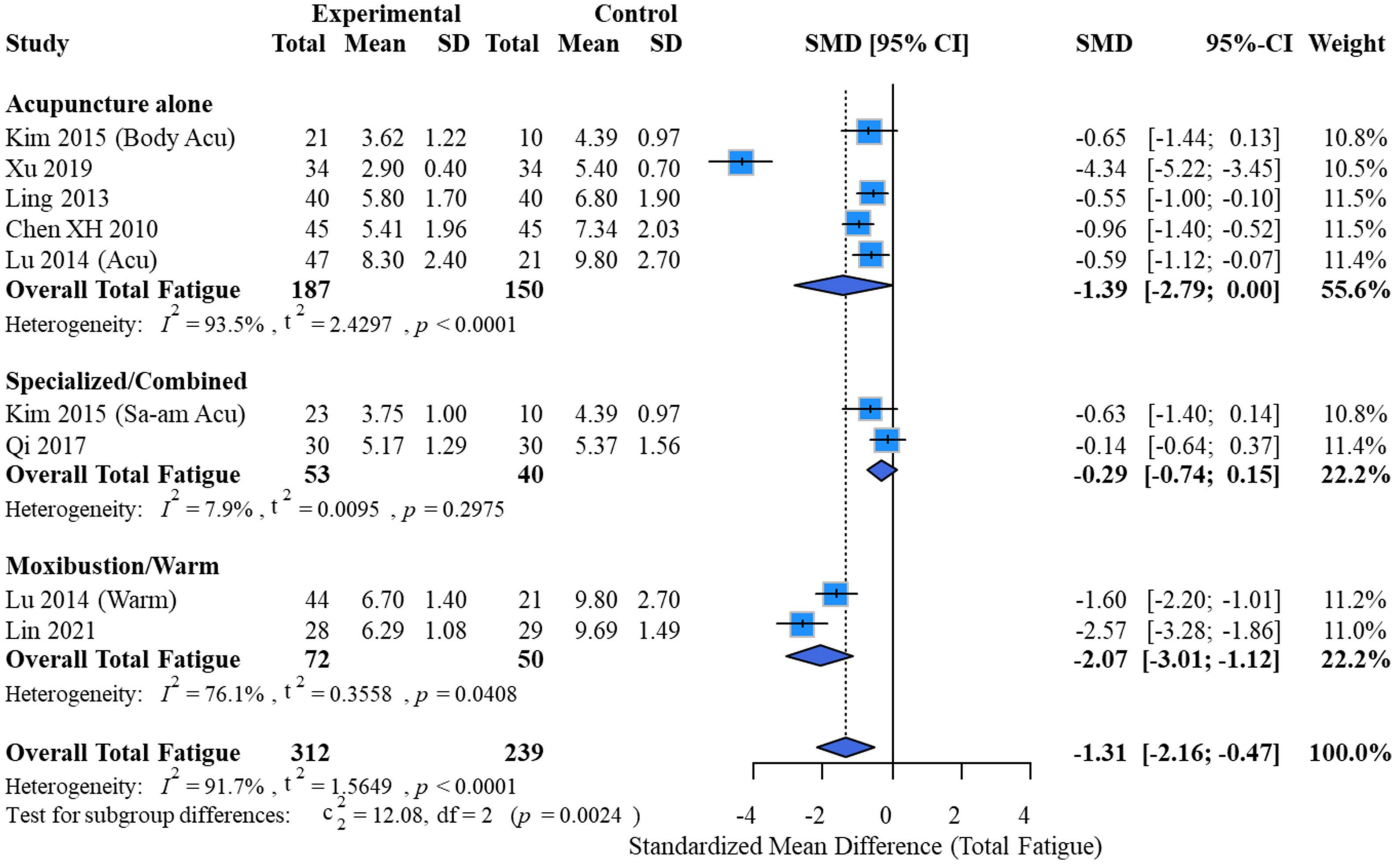
Forest plot of the meta-analysis for overall fatigue score.

Further subgroup analysis identified intervention type as a potential source of variation (*p* = 0.0024). Warm-needling/moxibustion yielded the greatest clinical benefit (SMD = −2.07, 95% CI: −3.01 to −1.12), while simple acupuncture (SMD = −1.39, 95% CI: −2.79 to −0.00) and combined acupuncture (SMD = −0.29, 95% CI: −0.74 to 0.15) did not reach statistical significance. Control types varied widely, including usual care (SMD –0.85), oryzanol + vitamin B1 (SMD –1.10), prednisone (SMD –1.50), non-acupoint acupuncture (SMD –0.65), sham acupuncture (SMD –0.92), Chinese herbal medicine alone (SMD –0.78), and lifestyle intervention (SMD –0.80). As effect sizes differ substantially across control types, pooling all controls together may obscure specific vs. non-specific effects and limits the clinical interpretability of the overall pooled estimate. Despite this, high heterogeneity persisted within subgroups (simple acupuncture I^2^ = 93.5%, warm-needling/moxibustion I^2^ = 76.1%), and other potential sources of heterogeneity—such as diagnostic criteria, intervention duration, control type, and baseline severity—were not formally explored, limiting the stability and interpretability of the results. Forest plots for these outcomes are illustrated in Figure 3.

**Figure 4:**
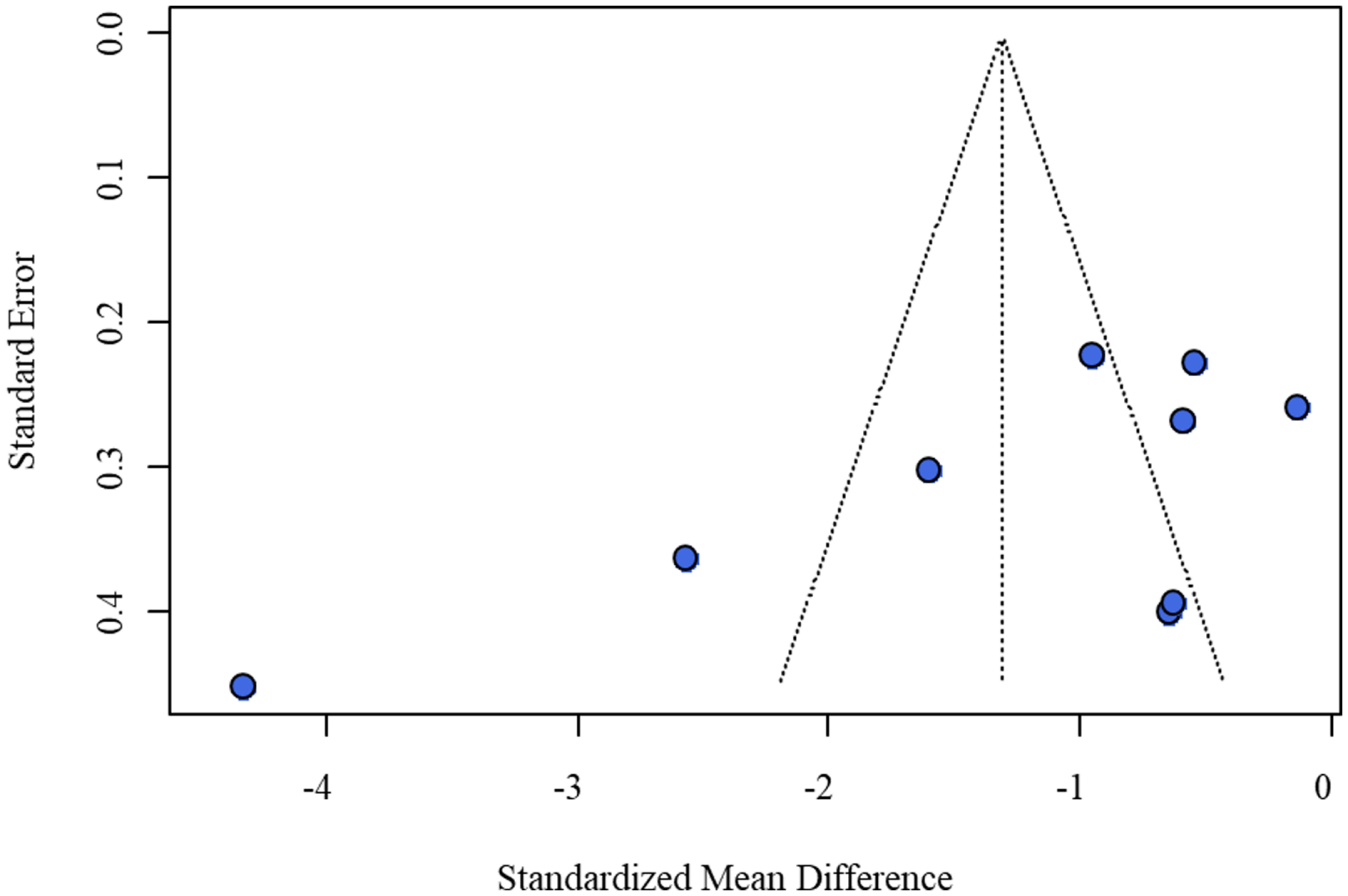
Funnel plot of the meta-analysis for overall fatigue score.

Notably, formal statistical testing for publication bias was omitted because the limited number of studies (*n* < 10) restricts the reliability of such assessments. Instead, the stability of the evidence was visually appraised using a funnel plot (Figure 4).

#### 3.4.2 Physical fatigue outcome

A total of five comparisons (*n* = 363 patients; 206 intervention vs. 157 control) provided evidence for the physical fatigue outcome. Given the moderate inconsistency identified among studies (I^2^ = 63.8%, *p* = 0.026), a random-effects framework was pre-specified. Acupuncture interventions demonstrated a superior effect in reducing physical fatigue levels compared to controls (pooled SMD = −0.52, 95% CI: −0.88 to −0.16, *p* = 0.0048).

**Figure 5:**
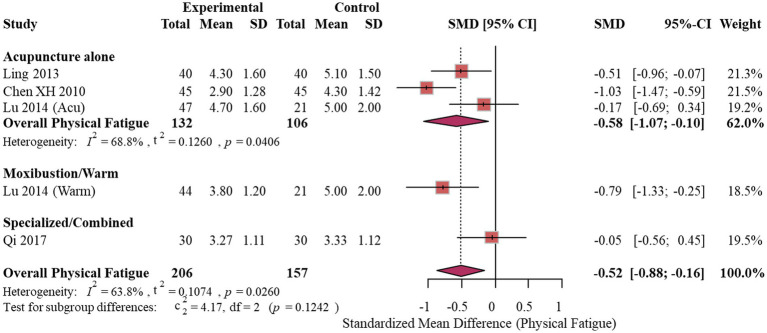
Forest plot of the meta-analysis for physical fatigue score.

Stratification by intervention modality revealed no significant heterogeneity between subgroups (*p* = 0.1242). In the categorical analysis, both simple acupuncture (SMD = −0.58, 95% CI: −1.07 to −0.10) and warm-needling/moxibustion (SMD = −0.79, 95% CI: −1.33 to −0.25) favored the intervention arm. However, the combination therapy subgroup failed to reach statistical significance (SMD = −0.05, 95% CI: −0.56 to 0.45). These findings are visualized in the Figure 5 forest plot.

**Figure 6:**
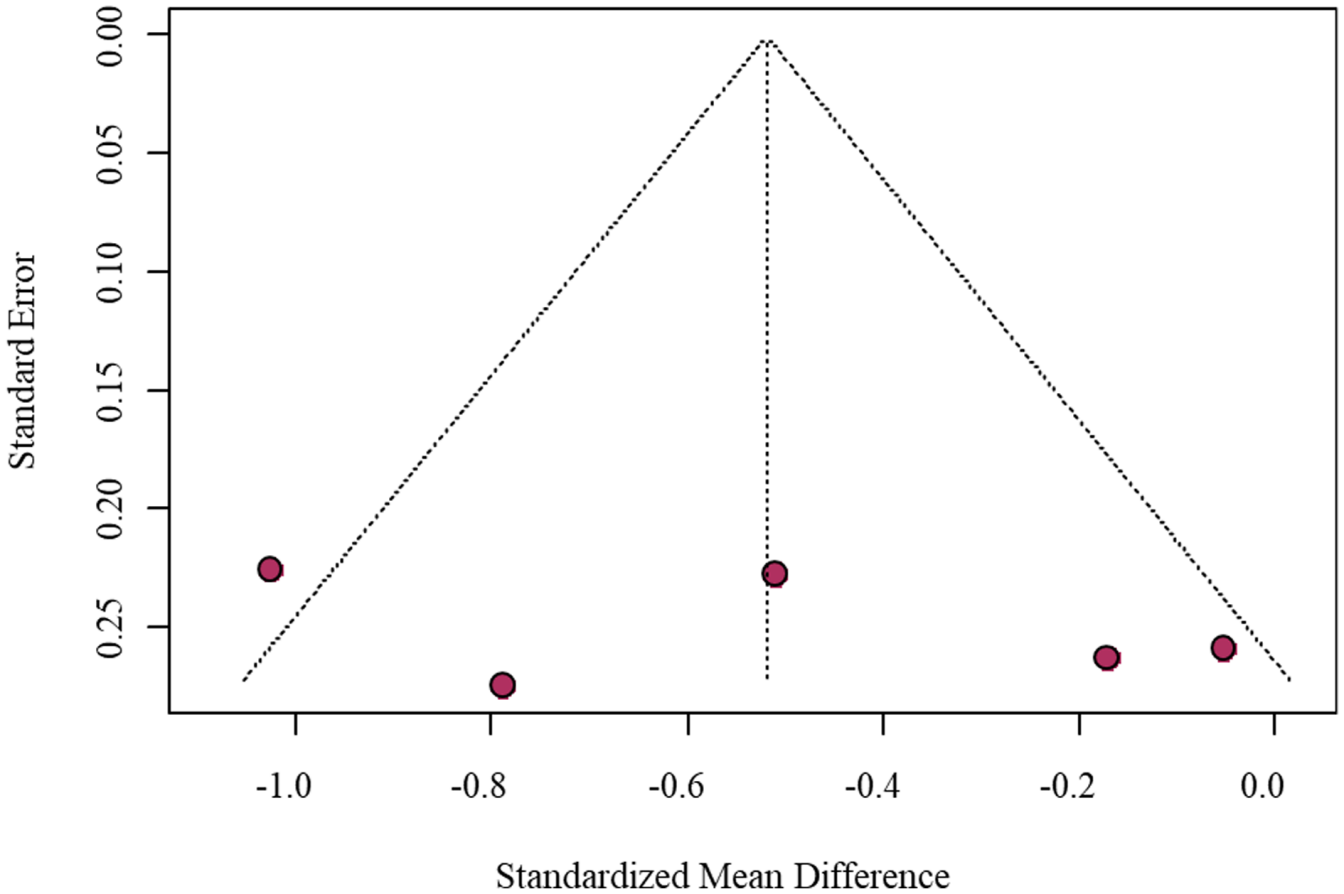
Funnel plot of the meta-analysis for physical fatigue score.

Again, because the number of comparisons was small (<10), no formal publication bias tests were performed; the funnel plot (Figure 6) was used only for descriptive assessment.

#### 3.4.3 Mental fatigue outcome

Mental fatigue outcomes were assessed using data from five comparisons (363 subjects; 206 in the intervention arm and 157 in the control arm). Based on a moderate heterogeneity level (I^2^ = 56.5%), the effect sizes were aggregated using a random-effects model. The results indicated that acupuncture treatments were superior to control interventions in reducing mental fatigue scores (pooled SMD = −0.59, 95% CI: −0.93 to −0.26, *p* = 0.0006).

**Figure 7:**
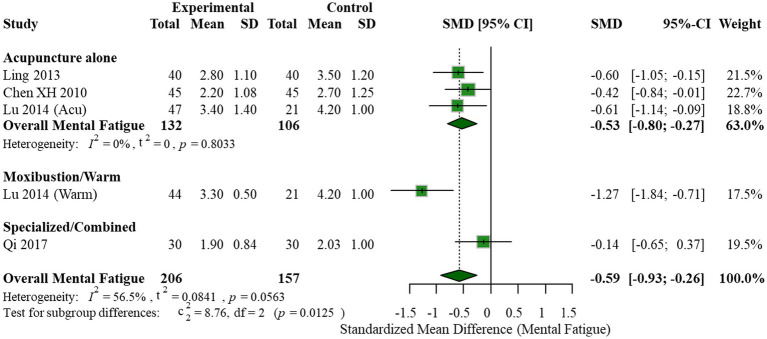
Forest plot of the meta-analysis for mental fatigue score.

**Figure 8:**
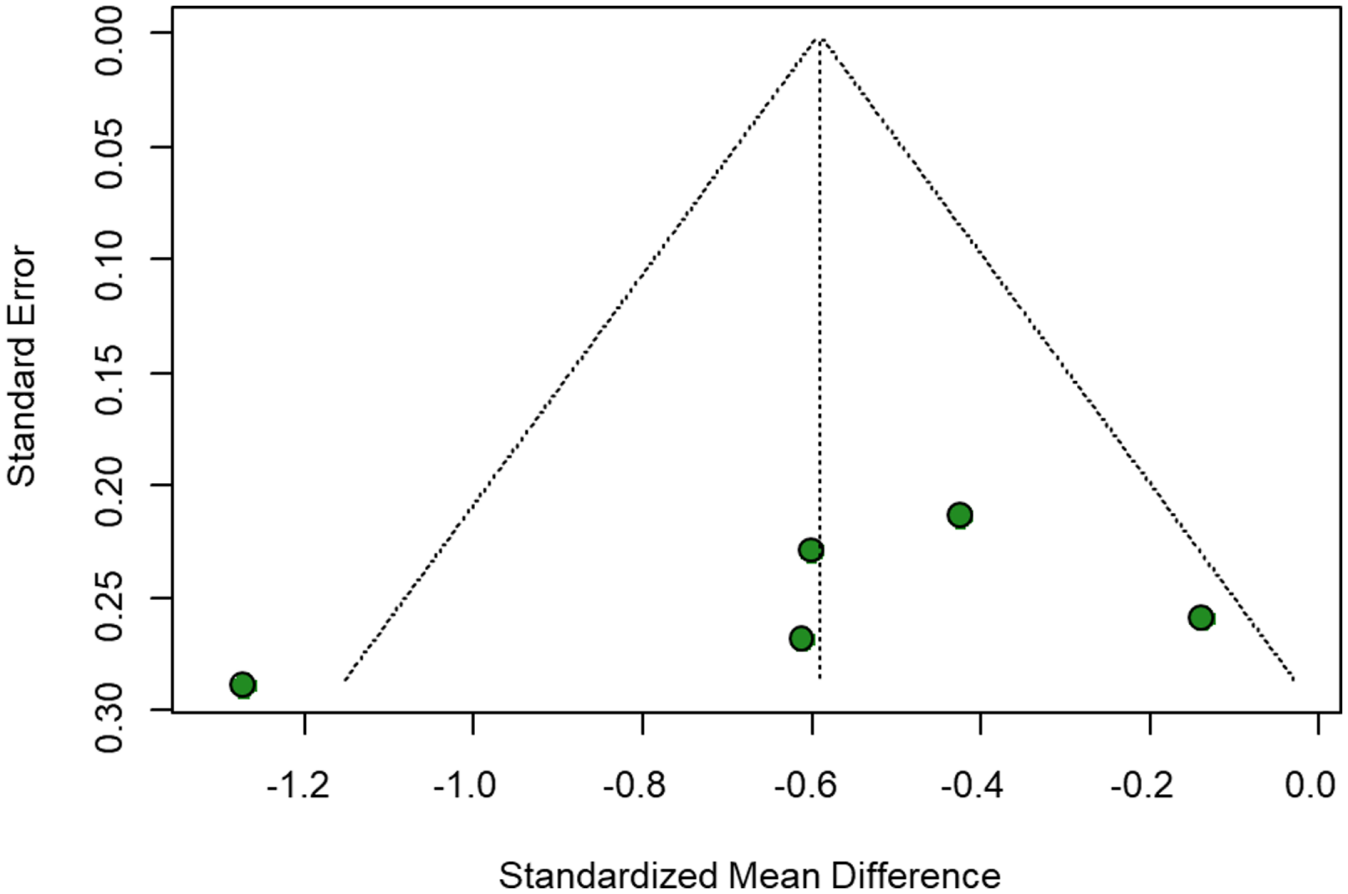
Funnel plot of the meta-analysis for mental fatigue score.

Differential effects were observed across intervention types during subgroup analysis (*p* = 0.0125). While simple acupuncture (SMD = −0.53) and warm-needling/moxibustion (SMD = −1.27) both significantly favored the intervention, the benefit was not sustained in the combined acupuncture subgroup (SMD = −0.14, 95% CI: −0.65 to 0.37). Figure 7 presents the forest plot for this outcome. As the number of comparisons was insufficient (*n* < 10) for robust publication bias detection, the funnel plot in Figure 8 was used for descriptive purposes only.

#### 3.4.4 Effectiveness and safety outcomes

Efficacy data, measured by total response rates, were aggregated from only two studies involving 152 participants, and adverse event data came from three studies. The intervention was associated with a higher clinical response rate (RR = 1.22, 95% CI: 1.07–1.40, *p* = 0.004); however, due to the small number of studies, small sample sizes, and wide confidence intervals, these results are highly unstable and should be considered exploratory. Notably, none of the included studies reported post-exertional malaise (PEM)-related data, which is the core diagnostic symptom and a key efficacy endpoint for CFS. Furthermore, most studies did not include follow-up assessments, precluding evaluation of the long-term efficacy of acupuncture. Safety assessments involving 338 patients did not identify explicit hazards, but the limited data and imprecise estimates warrant cautious interpretation.

**Figure 9:**
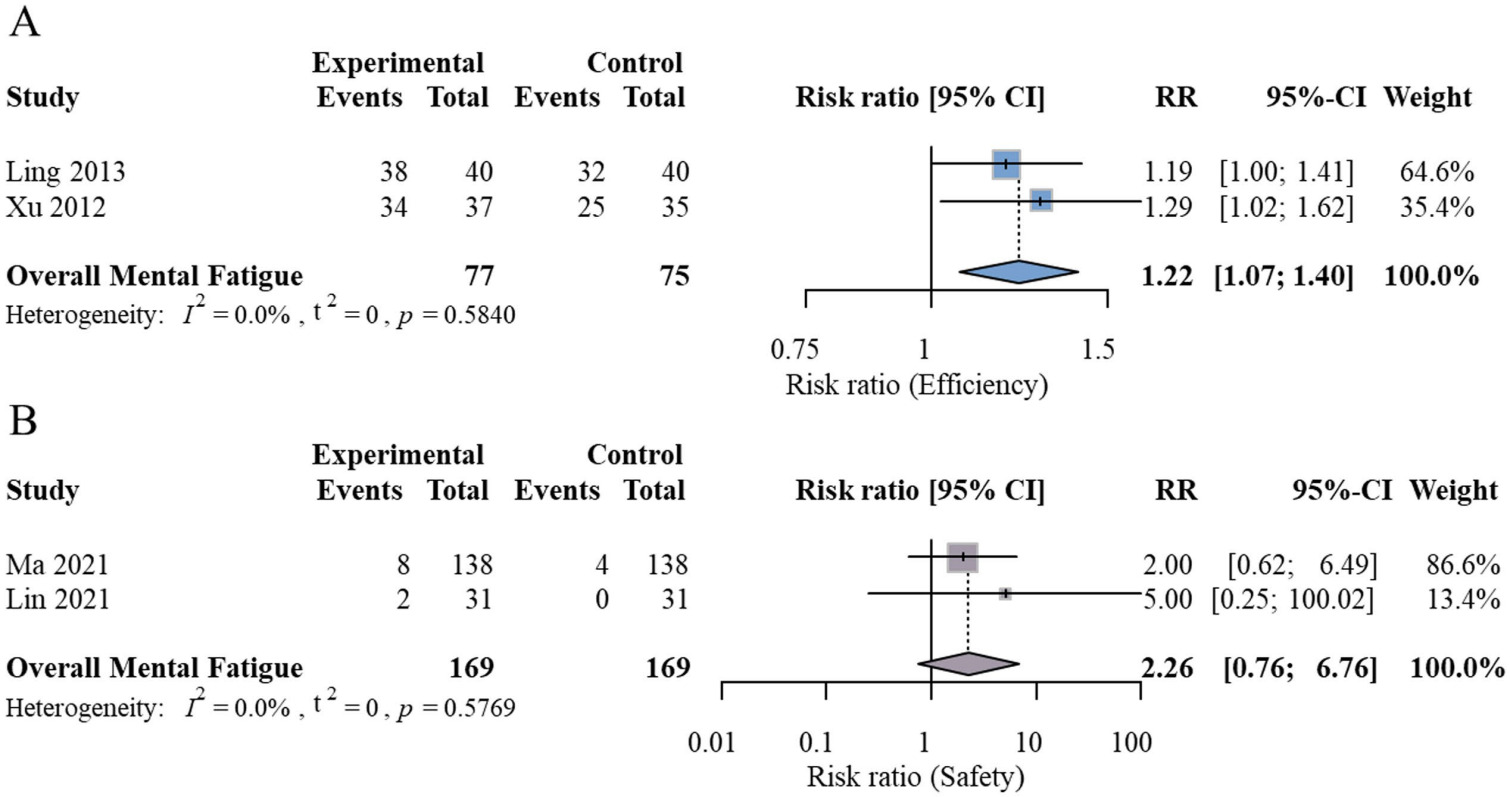
Forest plots of the meta-analysis for effectiveness and safety. Panel **A** represents the response rate, and panel **B** represents the adverse event rate.

Due to the small number of included trials and the relatively imprecise safety estimates (indicated by the broad confidence interval), the current results should be viewed as preliminary and interpreted with caution. Detailed forest plots illustrating these effectiveness and safety comparisons are available in Figure 9 (panels A and B).

**Figure 10:**
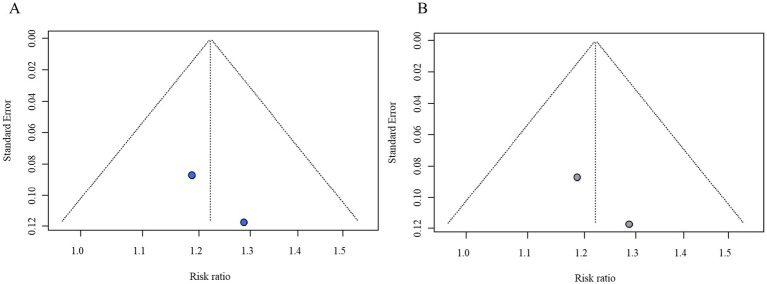
Funnel plots of the meta-analysis for effectiveness and safety. Panel **A** represents the response rate, and Panel **B** represents the adverse event rate.

As the number of included studies was less than 10, formal publication bias tests were not performed; the funnel plots (Figure 10A, panel A for response rate, panel B for adverse event rate) are provided for descriptive purposes only.

## 4 Discussion

In the present study, 8 RCTs with 715 participants were included to review available evidence on acupuncture-based treatments for fatigue symptoms and safety; given the limited and heterogeneous data, the findings are provisional. The findings indicated that, compared with control measures, acupuncture-related interventions produced statistically significant improvements in overall, physical, and mental fatigue. Furthermore, although based on a small number of studies, a favorable tendency was noted in the total response rate. No evidence emerged from the available data to suggest that these therapies meaningfully raise the risk of adverse events. Taken together, these findings suggest a potential benefit of acupuncture-related approaches, but given the limited evidence, they should be considered exploratory rather than definitive (16–20).

An important feature of this study is that it not only evaluated the overall fatigue outcome but also further analyzed the two dimensions of physical fatigue and mental fatigue. CFS is not a single fatigue state but a complex syndrome characterized by reduced physical tolerance, impaired cognitive function, insufficient recovery from sleep, and post-exertional worsening of symptoms (21, 22). Therefore, using only a single total score may not fully reflect the true clinical value of an intervention. The results of this study showed that acupuncture-related therapies tended to improve different fatigue dimensions, suggesting that their potential effects may not be limited to alleviating physical fatigue but may also regulate cognitive-related fatigue or central symptoms (23–25).

The findings of this study are generally consistent with previous systematic reviews on acupuncture and moxibustion for CFS. Prior reviews have commonly suggested that acupuncture or moxibustion may be superior to certain control interventions in improving fatigue scores and clinical response rates; however, they have also consistently highlighted issues such as small sample sizes, variable methodological quality, and substantial clinical heterogeneity in primary studies. For instance, Wang et al. (24) and Zhang et al. (25) concluded that acupuncture-related therapies may help relieve CFS symptoms, but the certainty of their conclusions was limited by insufficient blinding and inadequate reporting of randomization in many included studies. Similarly, the network meta-analysis by Fang et al. (26) and the systematic review on moxibustion by You et al. (27) reached analogous conclusions—namely, that acupuncture or moxibustion may have some efficacy, yet the overall level of evidence remains low. Thus, the current evidence base is more appropriate to support the judgment that “acupuncture-related therapies may be effective” rather than to draw high-certainty, definitive conclusions.

The subgroup analyses in the present study were exploratory and suggest that different acupuncture regimens may have variable effects; however, small numbers and protocol heterogeneity preclude definitive comparative conclusions. For overall fatigue and mental fatigue outcomes, the warm-needling/moxibustion subgroup showed a relatively more pronounced improvement trend, whereas the results for the simple acupuncture and combined therapy subgroups were less stable. These observations should be interpreted with caution. On the one hand, moxibustion and warm-needling combine acupoint stimulation with thermal stimulation, which might theoretically confer additional benefits for fatigue, sleep disturbances, and certain autonomic symptoms. On the other hand, the number of studies in each subgroup was small, and intervention protocols were highly variable; therefore, it is not yet possible to conclude that any one acupuncture technique has a clear advantage. These findings are more suitable as a reference for future trial design and protocol optimization rather than as a direct basis for clinical preference.

Importantly, the pooled analysis for overall fatigue exhibited extremely high heterogeneity (I^2^ = 91.7%), which persisted within subgroups (simple acupuncture I^2^ = 93.5%; warm-needling/moxibustion I^2^ = 76.1%). This heterogeneity likely arises from multiple sources, including variability in interventions (simple acupuncture, moxibustion, warm-needling, combined treatments) and substantial differences in control groups. The control interventions varied widely, encompassing usual care, oryzanol + vitamin B1, prednisone, non-acupoint acupuncture, sham acupuncture, Chinese herbal medicine alone, and lifestyle intervention. Effect sizes differ fundamentally across control types: for example, the SMD versus sham acupuncture (−0.92) better reflects specific efficacy, whereas the SMD versus usual care (−0.85) incorporates non-specific effects. The lack of stratified analysis by control type reduces the interpretability and clinical relevance of the pooled results. Moreover, other potential sources of heterogeneity—such as differences in fatigue assessment scales, diagnostic criteria, intervention duration, baseline symptom severity, and disease duration—were not formally explored.

Regarding safety, no significant difference in adverse event frequency was noted between the acupuncture and control arms, which is consistent with prior evidence (17, 28, 29). However, this conclusion is limited by the small volume of safety data and non-standardized reporting practices. Safety findings remain preliminary; the current evidence suggests that “no explicit safety hazards have been confirmed,” but caution is still warranted. Future investigations should prioritize systematic recording of side effects—ranging from minor subcutaneous hemorrhages to post-treatment fluctuations—to enable a more transparent safety evaluation.

Several limitations of this study must be acknowledged. First, the methodological quality of the primary studies is a major concern. Among the eight included RCTs, only one was at low overall risk of bias, seven raised “some concerns,” and one was at high risk. Six studies inadequately described random sequence generation and allocation concealment; only one implemented blinding, the remainder were open-label, and none blinded outcome assessors; six studies were not pre-registered, posing a risk of selective reporting. These flaws may have led to overestimation of effect sizes. Given that fatigue assessments rely heavily on subjective patient feedback, the potential for placebo effects remains a significant concern. Consequently, the statistical significance observed in this meta-analysis warrants cautious interpretation.

Second, the number of included RCTs is small, with a correspondingly modest total sample size (*n* = 715). Third, the evidence is geographically concentrated, as nearly all studies originated from China (one from Korea), which may limit the generalizability of our conclusions to broader clinical contexts. Fourth, clinical heterogeneity across studies was substantial, undermining the stability of the pooled estimates. Fifth, most studies reported only short-term outcomes and lacked medium- and long-term follow-up; therefore, this review could not evaluate the sustained benefits of acupuncture-related therapies. Sixth, certain outcomes—including total response rate (only two studies) and adverse events (only three studies)—were reported in very few trials, with small sample sizes and wide confidence intervals, rendering the results highly unstable. Most critically, none of the included trials reported data on PEM, a core diagnostic symptom and a key efficacy endpoint for CFS. The absence of follow-up data further precludes any assessment of long-term efficacy. Consequently, the conclusions of this study should be understood as a provisional synthesis of the limited available evidence rather than as definitive findings.

## 5 Conclusion

Current limited evidence suggests that acupuncture-related therapies may have potential benefits for overall, physical, and mental fatigue in CFS patients; however, these findings are highly provisional and should be interpreted with caution due to the small number of studies, small sample sizes, moderate risk of bias, high heterogeneity, and lack of standardized reporting. Specific recommendations regarding which type of acupuncture, optimal duration and frequency of intervention, patient selection criteria, and the role of acupuncture within comprehensive CFS management cannot be made at this stage. Future high-quality, multicenter RCTs with uniform diagnostic criteria, clearly defined intervention protocols, adequate follow-up, and standardized outcome assessment are required to determine the efficacy, safety, and clinical applicability of acupuncture-related therapies in CFS.

## Data Availability

The original contributions presented in the study are included in the article/supplementary material, further inquiries can be directed to the corresponding author.
